# Fungal Communities Vectored by *Ips sexdentatus* in Declining *Pinus sylvestris* in Ukraine: Focus on Occurrence and Pathogenicity of Ophiostomatoid Species

**DOI:** 10.3390/insects12121119

**Published:** 2021-12-14

**Authors:** Kateryna Davydenko, Rimvydas Vasaitis, Malin Elfstrand, Denys Baturkin, Valentyna Meshkova, Audrius Menkis

**Affiliations:** 1 Department of Forest Mycology and Plant Pathology, Uppsala BioCenter, Swedish University of Agricultural Sciences, P.O. Box 7026, SE-75007 Uppsala, Sweden; kateryna.davydenko74@gmail.com (K.D.); rimvys.vasaitis@slu.se (R.V.); malin.elfstrand@slu.se (M.E.); 2 Ukrainian Research Institute of Forestry & Forest Melioration, Pushkinska St. 86, 61024 Kharkiv, Ukraine; valentynameshkova@gmail.com; 3 State Forest Protection Service “Kharkivlisozahyst”, Nezalezhnosti 127, Pokotilovka, 62458 Kharkiv, Ukraine; baturkin.denis@ukr.net

**Keywords:** *Ips sexdentatus*, *Pinus sylvestris*, ophiostomatoid fungi, insect-fungal-tree interactions, forest dieback, drought, climate change

## Abstract

**Simple Summary:**

Bark beetles serve as vectors to numerous tree pathogens, the most conspicuous guild of which are ophiostomatoid fungi. Most of these fungi are known to cause blue-stain discoloration of wood, and some of them are pathogenic to trees, in certain cases able to kill them. Over the last years, drought-induced stress and attacks by bark beetle *Ips sexdentatus* resulted in a massive dieback of *Pinus sylvestris* in Ukraine. Limited and fragmented knowledge is available as to which ophiostomatoid fungi in this geographic area are vectored by *Ips sexdentatus*, and their roles in tree dieback. It is known, though, that in different parts of Europe those fungal communities might significantly differ. This study represents the first and so far, the most extensive analysis of fungal associates of *I. sexdentatus* in eastern Europe accomplished combining different methods, using insect, plant, and fungal material, and reports a number of previously unknown insect-vectored pathogens of *P. sylvestris*. Increasing climate change-related disturbances to forests put reported findings in a broader geographical context.

**Abstract:**

Drought-induced stress and attacks by bark beetle *Ips sexdentatus* currently result in a massive dieback of *Pinus sylvestris* in eastern Ukraine. Limited and fragmented knowledge is available on fungi vectored by the beetle and their roles in tree dieback. The aim was to investigate the fungal community vectored by *I. sexdentatus* and to test the pathogenicity of potentially aggressive species to *P. sylvestris*. Analysis of the fungal community was accomplished by combining different methods using insect, plant, and fungal material. The material consisted of 576 beetles and 96 infested wood samples collected from six sample plots within a 300 km radius in eastern Ukraine and subjected to fungal isolations and (beetles only) direct sequencing of ITS rDNA. Pathogenicity tests were undertaken by artificially inoculating three-to-four-year-old pine saplings with fungi. For the vector test, pine logs were exposed to pre-inoculated beetles. In all, 56 fungal taxa were detected, 8 exclusively by isolation, and 13 exclusively by direct sequencing. Those included nine ophiostomatoids, five of which are newly reported as *I. sexdentatus* associates. Two ophiostomatoid fungi, which exhibited the highest pathogenicity, causing 100% dieback and mortality, represented genera *Graphium* and *Leptographium*. Exposure of logs to beetles resulted in ophiostomatoid infections. In conclusion, the study revealed numerous *I. sexdentatus*-vectored fungi, several of which include aggressive tree pathogens.

## 1. Introduction

Heat and drought affect plant chemical defenses as, e.g., susceptibility of trees to pests and pathogens, one conspicuous example for which are bark beetle attacks [1]. Consequently, undergoing climate warming, the currently experienced drought-induced reduction in tree vigor has contributed to increased tree mortality, becoming a widespread phenomenon on a continental scale [2]. For example, in conifer stands of central Europe, the acute drought has been reported to be an important driver of bark beetle infestation [3]. Yet another driver is beetle-vectored fungal symbionts, which help the beetles in nutrient acquisition and detoxification of toxic tree secondary metabolites, thus furthermore weakening tree defense mechanisms, thereby aiding in a successful beetle attack [4,5].

Six-toothed bark beetle (*Ips sexdentatus*) (Börner, 1767) (Coleoptera: Curculionidae) is a secondary pest of pines, attacking weakened trees, but following significant climatic disturbances, such as wildfires, drought, and windstorms, extensive outbreaks of this insect can occur over large forest areas, resulting in massive tree mortality, economic losses, and ecological impacts. To date, the most extensive attacks on living trees by *I. sexdentatus* have been reported from pine stands of south-western Europe [6,7,8,9]. For secondary forest pests, such as *I. sexdentatus*, global warming is predicted to increase the number of generations and larger broods, thus the resulting increase in population-level could trigger more frequent outbreaks, and on a broader geographic scale [9].

Little is known regarding the role and impact of *I. sexdentatus* in forest dieback in other parts of Europe. An interesting situation from this point of view has developed in Ukraine in recent years. Since 2010, due to drought-induced stress and bark beetle attacks, a massive dieback of *Pinus sylvestris* (L.) is being observed in eastern, northern, and central parts of the country, covering a total area of about 70,000 ha, and *I. sexdentatus* is preliminary reported as one of the most important damage agents, along with pine engraver beetle (*Ips acuminatus*) (Gyllenhal, 1827) (Coleoptera: Curculionidae) [10]. In this context, the role of *I. sexdentatus* as a dieback agent of pine plantations of Ukraine requires a more detailed investigation. The geographic area is of particular interest as it is located at the south-eastern edge of the natural distribution of *P. sylvestris* in Europe [11].

As the most bark beetles, *I. sexdentatus* are associated with specific fungi, so-called ophiostomatoid or blue-stain fungi (e.g., genera *Ophiostoma*, *Leptographium*, *Graphium*, *Ceratocystis*, etc.), in numerous cases reported to be aggressive, even lethal tree pathogens [12]. To date, fungal associates of *I. sexdentatus* have been reported from Poland [13], Fennoscandia [14,15], France [16,17], and Spain [8,18], in all revealing associations of *I. sexdentatus* with over ten ophiostomatoid species. Yet, no data on the fungal associates of *I. sexdentatus* are available from the eastern area of insect distribution. However, there are certain indications that the species composition of ophiostomatoids in different geographic areas differ. For example, Lieutier et al. [17] reported that “*Ophiostomales* associated with *I. acuminatus* in south-eastern France included four species that differed greatly from the Scandinavian and German flora associated with the same insect”.

Notably, all cited studies have been focused on ophiostomatoid fungi. More comprehensive studies of the overall fungal community associated with *I. sexdentatus* in declining pine trees are not yet available, even though they might provide valuable information regarding the presence of other potential pathogens and/or decay fungi, suggesting implications on the future health of invaded forest stands, as well as the wood quality of infested stems. For example, in this respect Bezos et al. [19] detected pine canker-/dieback-causing fungi as *Diplodia sapinea* (Fr.) Fuckel and *Fusarium circinatum* Nirenberg & O’Donnell in *I. sexdentatus* galleries collected from baited trap logs. Moreover, previous studies of pine bark beetles *I. acuminatus* and *Hylurgus ligniperda* (Fabricius, 1787) (Coleoptera: Curculionidae) conducted in eastern Ukraine [20,21] detected the presence in adult beetles of a number of wood-decay basidiomycetes: *Bjerkandera adusta* (Willdenow) P.Karsten, *Fomitopsis pinicola* (Swartz) P.Karsten, *Phlebiopsis gigantea* (Fries) Jülich, but also *Heterobasidion annosum* (Fries) Brefeld,—root and stem decay fungus of primary economic importance.

Herewith, we hypothesize that: (1) communities of *I. sexdentatus*-vectored fungi in south-west and south-east Europe differ; (2) in the south-east, they include some yet unknown tree pathogens, some of which might represent alien invasive species; (3) apart from ophiostomoids, *I. sexdentatus* vectors a broad range of fungi, representing distinct ecologic and systematic guilds; (4) combining different methods, e.g., fungal pure culture isolations vs. direct DNA analysis provides a synergistic effect in analysis of fungal communities. The aims of this study were to: (1) investigate fungal communities associated with *I. sexdentatus* in Ukraine in the context of available related European reports, focusing on ophiostomatoid fungi (2) test pathogenicity of ophiostomatoid fungi to *P. sylvestris*, and (3) check whether Leach’s postulates can be met owing to confirm *I. sexdentatus* as their vector. Leach [22] has suggested four postulates to confirm that an insect is a vector for pathogens that cause the disease to plants as follows: (1) a close association between the insect and diseased plants; (2) regular visits by the insect to healthy plants; (3) the presence of the pathogen on the insect in nature; (4) transmission of the pathogen to the host under controlled conditions.

## 2. Materials and Methods

### 2.1. Collection of Insects

**Figure 1:**
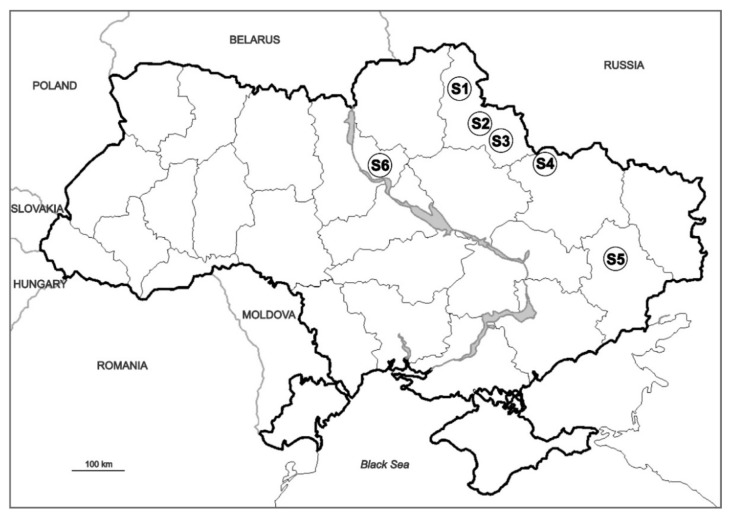
Map of Ukraine showing study sites denoted by S1–S6.

In September 2016, adults of *I. sexdentatus* and infested bark, phloem, and sapwood were collected in six 50–60-year-old pure plantations of *P. sylvestris* situated in northern, central, and eastern Ukraine (Figure 1). In each of the stands, between 25% and 75% of the growing stock exhibited decline symptoms, namely trees being severely weakened by drought and bark beetle attacks. In total, 576 beetles (96 per site) were collected by removing them from galleries and individually placing them into sterile 1.5 mL centrifugation tubes. The time of sampling coincided with the end of the flying period of the bark beetles, and when the galleries under the bark have already been formed.

### 2.2. Sampling from Wood beyond Ips Sexdentatus Galleries

Ophiostomatoid fungi were isolated from the sapwood beyond breeding galleries of *I. sexdentatus*, following the protocol described by Solheim et al. [23]. Four trees per site, infested by *I. sexdentatus* were selected for sampling. Two woodblocks containing the galleries on the sapwood surface were cut from sections of a trunk from the lower part of standing trees at the height of 60–100 cm, and the same day transported to the laboratory. In the laboratory, two sapwood samples (approx. 18 mm diameter and 10 mm thick) from each of (debarked) woodblocks were taken 5–10 mm apart from the bark beetle gallery from the zone of visible blue-stain using a sterilized cork borer. In all, ninety-six sapwood samples from 24 trees (four trees per site) were subjected to fungal isolation.

### 2.3. Isolation and Morphological Grouping of Fungi

Isolations were made from beetles and wood samples. Half of the collected beetles (288 in total) were not surface sterilized, and from centrifugation, tubes were placed directly on 9 cm Petri dishes containing 3% malt extract agar (MEA) with antibiotic to select for *Ophiostoma* species. Samples of blue-stained wood beyond beetle galleries were surface-sterilized in 95% ethanol for 15 s, subsequently dried on a sterile paper towel, and placed on 3% MEA containing 200 ppm of cycloheximide and 300 ppm of streptomycin (Sigma-Aldrich, MO, USA) to avoid the growth of bacteria and fast-growing fungi. The plates were incubated at 22 °C in the dark and were checked daily. Actively growing fungal colonies were sub-cultured on 3% MEA without antibiotics and grouped according to mycelial morphology using both a stereomicroscope and a fluorescence microscope Olympus BX51 (Olympus America, Inc., New York, NY, USA) after anamorph fruiting structures were mounted on glass slides and stained in cotton blue.

### 2.4. DNA Extraction, Amplification and Sequencing

The material included: (1) pure cultures of isolated fungi; (2) collected adult beetles. First, one isolate per each group of fungal cultures was used for DNA extractions, amplification, and Sanger sequencing following methods described by Menkis et al. [24]. Amplification by PCR was done using ITS1F [25] and ITS4 [26] primers. The thermal cycling was carried out using an Applied Biosystems GeneAmp PCR System 2700 thermal cycler (Foster City, CA, USA). DNA was amplified in a 10 µL reaction mixture containing 0.25 µL of DreamTaq DNA Polymerase 5 U/µL (Thermo Scientific™, West Sacramento, CA, USA), 1 µL of 10 × DreamTaq Green Buffer, 0.5 µL of dNTP Mix (2 mM each #R024), 0.3 µL of each primer (25 µM), 1 µL (5 ng/µL) template DNA and water, nuclease-free (#R0581) up to 10 µL. An initial denaturation step at 95 °C for 5 min was followed by 35 amplification cycles of denaturation at 95 °C for 30 s, annealing at 55 °C for 30 s, and extension at 72 °C for 30 s. The thermal cycling was ended by a final extension step at 72 °C for 7 min. PCR products were size separated on 1% agarose gels and visualized under UV light. Sequencing was carried out by Macrogen Inc., Korea. Raw sequence data were analyzed using the SeqMan Pro version 10.0 software from DNASTAR (DNASTAR, Regent St. Madison, WI, USA). Sequences were identified using BLASTn and GenBank nucleotide database (https://blast.ncbi.nlm.nih.gov/Blast.cgi, accessed on 25 October 2021). The criteria used for identification were sequence coverage >80%; similarity to taxon level 98–100%, similarity to genus level 94–97%.

Second, isolation of DNA from not surface-sterilized adults of bark beetles was done from 288 individuals. Isolation of DNA was done from each bark beetle separately using the CTAB protocol [24]. Amplification and sequencing of fungal ITS rDNA were carried out as previously described, performing amplification by PCR in two steps: (1) using fungal specific primers NLC2 and NSA3; (2) nested PCR using primers ITS1F and ITS4 [27]. PCR products were size separated on 1% agarose gels and visualized under UV light. If only one DNA band was present on the gel per sample, following nested PCR, the PCR product was used for sequencing. Multiple-banded PCR products (indicating the presence in a sample of more than one fungal species) were separated on 2.0% agarose gels and individual bands were re-amplified using universal primers ITS1 and ITS4. The resulting single-band products were sequenced in both directions using the same primers as for PCR amplification. Sequencing and analysis of sequencing data were performed as described above.

### 2.5. Pathogenicity Tests

To study the potential impact of fungi associated with *I. sexdentatus* on *P. sylvestris* trees, inoculation tests were conducted using 3–4-year-old saplings of *P. sylvestris*. Six isolates obtained during the present work, each representing a distinct taxon of ophiostomatoid fungi, were used: *Graphium* sp. KD5, *Grosmania penicillata*, *Leptographium olivaceum, Leptographium sosnaicola, Ophiostoma bicolor*, and *Ophiostoma canum*. A total of 72 saplings were inoculated with six isolates representing each of the taxa (12 trees per isolate/taxon). Inoculation was done by removing ca. 10 mm x 15 mm bark area on the stem using a sterile scalpel, placing MEA plugs with actively growing fungal mycelia as an inoculum on the sapwood, covering it up with the bark, and then wrapping around the stem using the Parafilm^®^ M (Merck KGaA, Darmstadt, Germany), as previously described by Krokene and Solheim [28]. For controls, twelve saplings were inoculated with sterile MEA plugs. The health status of the saplings was checked weekly for 6 months. After 6 months, all plants were harvested, eventual symptoms recorded and, if present, the size of the necrotic lesion was measured.

### 2.6. Vector Test

Leach’s postulates [29,30] were tested to confirm whether *I. sexdentatus* is a vector for two selected ophiostomatoid taxa, *O. minus*, and *Graphium* sp. KD5. Postulates 1 and 2 were positively tested in a survey of *P. sylvestris* stands for the presence of *I. sexdentatus* (while assigning study plots), as the beetle has been frequently observed both on healthy-looking trees and trees showing dieback symptoms. Postulate 3 (presence of *O. minus* and *Graphium* sp. KD5 on *I. sexdentatus* in nature) was positively tested while accomplishing study work for isolation and identification of fungi from the beetles (described in Section 2.1, Section 2.3 and Section 2.4).

To test Postulate 4, a total of sixty living adult beetles of *I. sexdentatus* were collected in the forest. In the laboratory, twenty of them were inoculated with *O. minus*, twenty with *Graphium* sp. KD5 (by spraying spore suspension on beetles), and twenty (harboring naturally acquired fungi) were allocated to be used for control infestations. The 10^5^ conidiospore/mL suspension, harvested by washing away the surface of fungal pure culture colonies with distilled water, was used for inoculations with each respective fungus. Artificial infestations with the beetles were accomplished by placing them on thirty approx. 18–22 cm diameter, 80–90 cm long freshly cut *P. sylvestris* logs. Ten logs were exposed to bark beetles inoculated with *O. minus*, ten with *Graphium* sp. KD5 and ten with non-inoculated beetles (controls), thus using two beetles per log. The logs were placed in aerated plastic containers (one log per container-sized 120 × 140 × 165 cm) and incubated for 30 days. To avoid desiccation, the ends of the logs were dipped into paraffin wax. After the incubation period, all logs were visually checked for symptoms of blue-stain fungi by debarking sapwood around the entry holes and visual checking the blue staining. To re-isolate *O. minus* and *Graphium* sp. KD5 and other ophiostomatoids, from logs showing *I. sexdentatus* entry holes, frass, and dust on the bark, as well as typical signs of maturation feeding (nibbling, girdling, or pruning), three pieces of wood tissue (in total 90 samples) approx. 1 × 1 cm in size were cut off and placed onto MEA containing antibiotics [29]. Similarly, 30 wood samples were subjected to fungal isolation from logs that were not exposed to *I. sexdentatus*.

### 2.7. Statistical Analyses

Chi-square and Kruskal Wallis tests, and the Sørensen similarity index were calculated using Minitab v. 18.1 (University Park, PA, USA).

## 3. Results

### 3.1. Fungal Communities Associated with Ips Sexdentatus

Fungal growth was observed from 97.9% of the beetles, yielding a total of 432 distinct isolates. Grouping of the isolates based on their culture morphology resulted in 42 morphotypes, assigned to respective taxon following ITS rDNA sequencing. Direct ITS rDNA sequencing of the fungal ITS rDNA from beetles resulted in 633 sequences representing 49 fungal taxa. When pooled, both direct amplification and fungal pure culture isolations from beetles resulted in 56 fungal taxa encompassing three fungal phyla: Ascomycota (forty-one fungal taxa including nine ophiostomatoid of fungi), Basidiomycota (five taxa), Mucoromycotina (four taxa), while six taxa remained unidentified (Table 1). The most commonly detected fungi by direct sequencing from the beetles were *Entomocorticium* sp. (12.3%), *Cladosporium* sp. (11.1%), and *Ophiostoma ips s.l.* (9.0%). There were eight taxa detected exclusively by pure culture isolations, 13 taxa detected exclusively by direct sequencing, and 35 taxa using both methods (Table 1). In the overall fungal community, the species diversity, revealed by each method was to a moderate extent similar (the Sørensen similarity index = 0.48) although direct sequencing had revealed more taxa (48 vs. 43) than pure culture isolation (*p* ≤ 0.05).

The ITS rDNA sequence analysis revealed that the ophiostomatoid fungi reside within three genera in two orders: *Leptographium sensu lato* and *Ophiostoma s. l.* in the Ophiostomatales, and *Graphium* in the Microascales (Table 1). Most commonly encountered were taxa of *Ophiostoma s. l.* (5 taxa: *Ophiostoma bicolor*, *O. canum*, *O. ips*, *O. minus* and *O. piceae*), followed by three *Leptographium s. l.* (*Grossmania penicillata*, *Leptographium olivaceum*, and *L. sosnaicola*), and one *Graphium* sp. *s. l*.

Out of 96 wood samples taken beyond *I. sexdentatus* galleries, 87 (90.1%) yielded growth of ophiostomatoid fungi, representing seven taxa. The taxa (species *sensu lato*) were *O. ips*, isolated from 63.5% sampled galleries, *O. minus* (48.9%), *O. bicolor* (38.5%), *Leptographium* *olivaceum* (30.2%), *L. sosnaicola* (27.1%), *O. canum* (27.1%), and *G. penicillata* (25.0%). Each of them was also detected in *I. sexdentatus* both by pure culture isolations from the beetle body and direct DNA sequencing (except for *O. bicolor* detected in beetles exclusively by isolation). Two taxa, *Graphium* sp. KD5 and *O. piceae*, that were both isolated and sequenced from beetles (Table 1), were not isolated from the wood. The overall abundance of fungal species as compared among different sites did not differ significantly (Kruskal Wallis, KW = 1.79, *p* = 0.62). Similarities in fungal community structures (as detected by the Sørensen index of qualitative similarity) in each of comparisons between the study sites (S1–S6) were low to moderate (Table 2).

### 3.2. Pathogenicity

Results of pathogenicity tests are presented in Table 3. Six months following inoculation, all tested fungi, except for *O. canum*, caused dieback symptoms and/or death of *P. sylvestris* saplings, although in different proportions (Table 3). Dieback symptoms included resin flow, needle discoloration, and wilt. *L. sosnaicola* and *Graphium* sp. KD5 were the most pathogenic, causing mortality to 75.0% and 58.3% of the saplings, and the dieback symptoms for the rest. *G. penicillata, L. olivaceum*, and *O. bicolor* caused mortality to 33.3%, 16.7%, and 8.3% of saplings, respectively (Table 3). Control saplings did not show symptoms of dieback. Lesions on stems were induced by each tested fungus. Depending on species, average lesion length varied between 3.4–25.9 mm, and in each interspecific comparison (except for *G. penicillata* & *O. bicolor*) the differences between means were statistically significant (Table 3). *L. sosnaicola* and *Graphium* sp. KD5 induced the largest lesions. Following the experiment, all inoculated fungi were in 62.5–100% of cases re-isolated (Table 3).

### 3.3. Vector Test

Entry holes and maturation feeding by adults of *I. sexdentatus* were observed on 10 out of 10 logs that were exposed to beetles inoculated with *O. minus*, in 9 out of 10 logs that were exposed to beetles inoculated with *Graphium* sp. KD5, and in 9 out of 10 logs exposed to non-inoculated beetles. *O. minus* was the only ophiostomatoid detected in logs exposed to its pre-inoculated *I. sexdentatus*, growing from 23 of 30 samples (76.7%). Similarly, *Graphium* sp. KD5 was the only ophiostomatoid detected in logs exposed to its pre-inoculated *I. sexdentatus*, growing from 26 of 30 samples (86.7%). Fungal isolations from control logs (exposed to non-inoculated beetles harboring naturally acquired fungi) yielded isolates of ophiostomatoid fungi from 27 (90%) of 30 samples. Here, five samples (16.7%) had growth of *O. minus*. Other fungi isolated from control logs were *O. canum, O. ips*, and *O. piceae*. *Graphium* sp. KD5 from control logs was not isolated. Differences in frequency of occurrence of both *O. minus* and *Graphium* sp. KD5 in logs exposed to their inoculated beetles vs. their occurrence in control logs were statistically significant (*p* = 0.002). Ophiostomatoid fungi were not isolated from the logs that were not exposed to *I. sexdentatus*.

## 4. Discussion

### 4.1. Ophiostomatoid Fungi

The ITS rDNA sequence data is insufficient to delineate species in some *Ophiostoma* species clusters [31]. Therefore, the authors were able to identify the species only to *sensu lato* level, which is a certain limitation of the present study. On the other hand, all available previous studies on *I. sexdentatus*-associated fungi focused on ophiostomatoid species and were also based on pure culture isolations from beetles, their galleries, and/or underlying sapwood, and the identifications were accomplished based on mycelial morphology and, in several instances, also by sequencing of ITS rDNA. This allowed us to make a comparative (yet with certain reservations) analysis with the results of preceding investigations. Thus, among the nine ophiostomatoids detected in the present work, the following four have been previously reported as associates of *I. sexdentatus*: *L.* *olivaceum* (as *Grosmania olivacea)* [15,18], *O. ips* [8,13,15,16,17,32], *O. minus* [13,15,18,33], and *O. piceae* [13,15]. This demonstrates the wide geographic distribution of the above-listed fungi. Moreover, considering above-cited studies, current work went even further, as sequencing of DNA was done also directly from the insect body. As a result, in addition, five *sensu lato* species herewith are for the first time presented as *I. sexdentatus* associates: *G. penicillata*, *O. bicolor*, *O. canum*, *Graphium* sp. KD5, and *L. sosnaicola*.

On the other hand, several ophiostomatoid fungi have been reported as associates of *I. sexdentatus*, which were not detected in the present work. For example, several *Sporotrix* spp. were reported as *I. sexdentatus* associates in north-western Spain [8], yet none of those fungi have been detected in the present Ukrainian study. Another, more recent example could be ophiostomatoids *Graphilbum furuicola* and *G. sexdentatum*, as reported from Norway [34]. This indicates that communities of the fungi in different geographic areas might differ to a significant extent, as has been previously noted [17]. This is also to some extent confirmed by the results of this work, as similarities in fungal community structures between our study sites, situated approx. 100–300 km apart, were low to moderate. In Poland, for example, (approx. 800–1300 km westwards from our study sites) a total of ten ophiostomatoid species have been isolated from *I. sexdentatus* beetles and galleries [13], only three of which (out of nine) were isolated during the present work, thus just about one-third in overlap. In Poland, *O. ips* was isolated from 35% of adult beetles and 44% of galleries, *O. minus* from 5% and 1%, *O. piceae* from 0% and 1% of beetles and galleries, respectively [13]. By contrast, in our study *O. minus* was isolated from 48.9% of galleries and was the second most common species.

### 4.2. Pathogenicity

Among the seven ophiostomatoids identified in the present study to the species level, six (*L. olivaceum*, *G. penicillata*, *O. bicolor*, *O. canum*, *O. ips*, *O. piceae*) in northern Europe are considered as species exhibiting “*low level of aggressiveness*”. In this respect, *O. minus*, which is regarded as “*relatively aggressive*” is an exception [15]. South European studies, however, demonstrated significant levels of aggressiveness for *O. ips* following its artificial inoculations to relatively large, 15–23 cm diameter (dbh) *P. sylvestris* trees [16,17,31]. Neither *O. ips* nor *O. minus* were not subjected to pathogenicity tests in the present work. In the previous studies, each of them was inoculated, respectively, to two-year-old [13] and three-year-old [21] seedlings of *P. sylvestris*. For *O. ips*, the first study reported mortality rates of 33.3%, which is in contrast with the results of the second study, where mortality was 0%. For *O. minus*, observed mortality incidences were, respectively, 100% [13] and 45% [21], here 25% of the plants exhibiting dieback symptoms. In all, the current work presents new data for pathogenicity for five ophiostomatoids vectored by *I. sexdentatus*.

Ophiostomatoid fungi, which in the inoculation tests exhibited the highest pathogenicity scores towards *P. sylvestris*, were *Graphium* sp. KD5 and *L. sosnaicola*, the latter of which has been recently detected in freshly felled *P. sylvestris* logs in Poland, and described as sp. nov. [35]. Notably, in pathogenicity tests both those fungi to the significant extent outscored all the rest of the species, and in all parameters investigated: (1) the incidence of lethal outcome; (2) the frequency of dieback symptoms; (3) the lesion length. On the other hand, the frequency of occurrence of *L. sosnaicola* and *Graphium* sp. KD5 on beetle bodies was scarce: the first was detected in 4.9% of the beetles, while the second,—in 2.6%. By contrast, their respective occurrence in the wood beyond galleries differed sharply—27.1% vs. 0%. Nevertheless, the latter, *Graphium* sp. KD5 was frequently isolated from wood samples from logs that were exposed to its pre-inoculated *I. sexdentatus* (in Section 3.3. *Vector test*). Reported findings, therefore, suggest that those fungi, along with *G. penicillata* and *L.* *olivaceum*, as well as previously reported *O. ips* and *O. minus* [13,21], play crucial roles in the dieback of *I. sexdentatus*-attacked pine. Further investigations are required to elucidate the identity and ecology of both fungi and constitute subjects for future work.

### 4.3. Other Fungi

As in the present work, certain widely spread wood-decay basidiomycetes, such as *Heterobasidion annosum, Fomitopsis pinicola, Phlebiopsis gigantea*, have been occasionally detected in previous related studies on bark beetles, including *Hylurgus ligniperda* [20], *I. acuminatus* [21], and *Ips typographus* [27]. Another basidiomycete, *Entomocorticium* sp., which might provide nourishment for *Dendroctonus* larvae [36], was detected in *I. sexdentatus* during the present work, and previously in *I. acuminatus* [21]. Characteristically, in both studies the fungus could have been detected only by direct DNA sequencing from the beetles, but not by mycelial isolations. It is known that the presence of *Entomocorticium* sp. may reduce the adverse effect on beetle larvae of their virulent fungus *O. minus* [36], providing larvae with protection and nutrients [12].

Among the detected ascomycetes, there were *A. pinea*, *B. fuckeliana*, *D. macrostoma*, *F. avenaceum*, *L. seditiosum*, *Nectria* sp., and *D. sapinea* that are potentially pathogenic fungi of *P. sylvestris*. The needle pathogen, *L. seditiosum*, and the root pathogen, *F. avenaceum*, were most frequently found in association with beetles. *Diplodia sapinea*, which is responsible for shoot blight and dieback of *P. sylvestris* trees, was isolated from 5.2% of the *I. sexdentatus* samples, although the fungus was previously reported in association with other pine beetles at a relatively high frequency [19,21]. This can probably be explained by *I. sexdentatus* not feeding in the crowns, where infection by *D. sapinea* typically takes place. Moreover, during the present work, no *Geosmithia* fungi were detected in *I. sexdentatus* in Ukraine, thus confirming the results of the previously conducted study [37].

### 4.4. Ips Sexdentatus as a Vector

Notably, each fungal species reported in the present study is indeed vectored by *I. sexdentatus*: each fungus has been found directly on/in a body of an adult beetle. Moreover, each ophiostomatoid that was found on a beetle body, has been also isolated from the wood beyond its galleries or entry holes. Although *Graphium* sp. KD5 and *O. piceae* were not isolated from wood during the community study (Section 3.1), yet in the vector test (Section 3.3) *Graphium* sp. KD5 was consistently isolated from wood beyond entry holes made by pre-inoculated *I. sexdentatus*, while *O. piceae* beyond entry holes made by the beetles after being naturally acquired by them in the forest. Two other species that were apparently acquired by *I. sexdentatus* under natural conditions and subsequently transferred to wood during the vector test were *O. canum* and *O. ips*.

## 5. Conclusions

To date, this is the most extensive study of fungal communities vectored by the bark beetle *Ips sexdentatus*. It provided clear positive/confirmative answers to each of the four study hypotheses drawn (see Introduction). The work included pure culture isolations in combination with direct DNA sequencing, and clearly demonstrated a synergistic effect in detection efficacy when both methods have been applied. As a result, proof has been provided that *I. sexdentatus* is a direct vector for numerous species of fungi representing a wide range of systematic and ecological guilds, for forest health ophiostomatoid fungi being the most important. The study demonstrated clear links between their occurrence on insect bodies, blue stain discoloration in infested wood, and pathogenicity. In all, nine species of ophiostomatoids have been detected, and five species are for the first time reported as *I. sexdentatus* associates. In all, the study provides new insights regarding the recent large-scale outbreak of *I. sexdentatus* (until recently regarded as a secondary forest pest), providing new insights into recently suggested bark beetle management methods and strategies, and addressing several of the pointed-out research needs [38].

## Figures and Tables

**Table 1:** Fungi in *Ips sexdentatus* beetles detected by direct ITS rDNA sequencing from insect bodies and by the sequencing of pure-culture mycelial isolates.

Taxa	Genbank	Detected in Beetles, % (No. Examined)		
	Accession No.	Direct Sequencing (288)	Isolations (288)	All (576)
**Ophiostomatoid** (species *sensu lato*)				
*Graphium* sp. KD5	OK576216	1.7	3.5	2.6
*Grosmannia penicillata*	OK576218	1.7	2.8	2.3
*Leptographium olivaceum*	OK576217	2.8	3.8	3.3
*Leptographium sosnaicola*	OK576219	4.9	4.9	4.9
*Ophiostoma bicolor*	OK576220	-	1.7	0.9
*Ophiostoma canum*	OK576221	5.9	2.4	4.2
*Ophiostoma ips*	OK576222	17.0	1.0	9.0
*Ophiostoma minus*	OK576223	7.6	1.4	4.5
*Ophiostoma piceae*	OK576224	1.0	0.7	0.9
**Other Ascomycota**				
*Alternaria alternata*	OK576225	1.4	1.4	1.4
*Anthostomella pinea*	OK576226	2.4	2.4	2.4
*Aspergillus versicolor*	OK576227	4.2	1.0	2.6
*Aureobasidium pullulans*	OK576228	2.8	2.4	2.6
*Beauveria bassiana*	OK576229	2.1	9.0	5.6
*Beauveria pseudobassiana*	OK576230	-	2.1	1.0
*Bionectria ochroleuca*	OK576231	6.6	2.8	4.7
*Botryotinia fuckeliana*	OK576232	9.4	3.1	6.3
*Chaetomium* sp.	OK576233	5.2	-	2.6
*Chalara* sp.	OK576234	2.1	2.1	2.1
*Cladobotryum mycophilum*	OK576235	5.9	-	3.0
*Cladosporium cladosporioides*	OK576236	12.5	-	6.3
*Cladosporium* sp.	OK576237	22.2	-	11.1
*Clavispora lusitaniae*	OK576238	2.1	-	1.0
*Cordyceps farinose*	OK576239	0.7	1.7	1.2
*Cyclaneusma minus*	OK576240	2.8	6.3	4.5
*Dactylonectria macrodidyma*	OK576241	3.8	2.1	3.0
*Diplodia sapinea*	OK576242	2.4	5.2	3.8
*Fusarium avenaceum*	OK576243	3.1	10.1	6.6
*Fusarium* sp.	OK576244	-	0.7	0.4
*Leptodontidium beauverioides*	OK576245	11.1	-	5.6
*Lophodermium seditiosum*	OK576246	2.1	11.1	6.6
*Mariannaea elegans*	OK576247	1.4	6.3	3.8
*Metapochonia bulbilosa*	OK576248	1.4	9.4	5.4
*Nectria* sp.	OK576249	2.8	-	1.4
*Penicillium citreonigrum*	OK576250	-	4.5	2.3
*Pezicula eucrita*	OK576251	0.7	1.4	1.0
*Phomopsis* sp.	OK576252	1.4	-	0.7
*Sydowia polyspora*	OK576253	1.4	3.5	2.4
*Talaromyces ruber*	OK576254	5.2	-	2.6
*Trichoderma asperellum*	OK576255	2.4	3.5	3.0
*Trichoderma* sp.	-	-	2.8	1.4
**Basidiomycota**				
*Entomocorticium* sp.	OK576256	24.7	-	12.3
*Filobasidium magnum*	OK576257	5.2	-	2.6
*Fomitopsis pinicola*	OK576258	0.7	8.3	4.5
*Heterobasidion annosum*	OK576259	5.6	0.3	3.0
*Phlebiopsis gigantea*	OK576260	3.1	1.0	2.1
**Mucoromycotina**				
*Mortierella gemmifera*	OK576261	-	1.7	0.9
*Mucor fragilis*	OK576262	5.9	-	3.0
*Mucor* sp.	-	-	1.7	0.9
*Umbelopsis isabellina*	OK576263	3.8	6.9	5.4
**Unidentified fungi**				
Fungal sp. A	OK576264	0.3	5.2	2.8
Fungal sp. HH78_19	OK576265	1.0	-	0.5
Fungal sp. K11	OK576266	1.7	3.8	2.8
Fungal sp. K21	OK576267	1.7	-	0.9
Fungal sp. K23	OK576268	0.3	2.1	1.2
Fungal sp. K27	OK576269	-	3.1	1.6
Total, no. (detected exclusively by the method)		48 (13)	43 (8)	56

**Table 2:** Similarities in fungal community structures between the study sites (Sørensen qualitative similarity index).

Site	S2	S3	S4	S5	S6
S1	0.37	0.38	0.37	0.37	0.38
S2		0.28	0.28	0.28	0.28
S3			0.26	0.26	0.26
S4				0.24	0.25
S5					0.23

**Table 3:** Pathogenicity tests with ophiostomatoid fungi (isolated from wood beyond *Ips sexdentatus* galleries and beetles) inoculated to 3–4-year-old saplings of *Pinus sylvestris*.

Fungus	Symptomatic Saplings, % (12 Tested per Fungus & Control)			Lesion Length, mm	Re-Isolation Frequency,
	Dead	Dieback Symptoms ^a^	All	(Mean ± SE) ^b^	%
*Graphium* sp. KD5	58.3	41.7	100	14.7 ± 1.2	87.5
*Grosmannia penicillata*	33.3	16.7	50	9.6 ± 1.9 A	87.5
*Leptographium* *olivaceum*	16.7	16.7	33.4	6.5 ± 0.2	91.7
*Leptographium sosnaicola*	75.0	25.0	100	25.9 ± 1.2	91.7
*Ophiostoma bicolor*	8.3	25.0	33.3	10.2 ± 0.3 A	100
*Ophiostoma canum*	0	0	0	3.4 ± 0.1	62.5
Control	0	0	0	0	0

^a^ Resin flow, needle discoloration, wilt. ^b^ Within the column differences between all means are statistically significant (*p* = 0.05) except for two followed by the letter A (*G. penicillata* & *O. bicolor*).

## Data Availability

Fungal ITS rDNA sequences generated during this study have been submitted to the GenBank and their accession numbers are provided in Table 1.
